# Classification of Hepatocellular Carcinoma Based on N6-Methylandenosine–Related lncRNAs Profiling

**DOI:** 10.3389/fmolb.2022.807418

**Published:** 2022-02-04

**Authors:** Lu Yin, Liuzhi Zhou, Shiqi Gao, Yina Feng, Hanzhang Zhu, Jingjing Xiang, Rujun Xu

**Affiliations:** ^1^ Department of Pathology, Affiliated Hangzhou First People’s Hospital, Zhejiang University School of Medicine, Hangzhou, China; ^2^ Department of Surgery, The Second Affiliated Hospital, Zhejiang University School of Medicine, Hangzhou, China; ^3^ Department of Neurosurgery, The Second Affiliated Hospital, Zhejiang University School of Medicine, Hangzhou, China; ^4^ School of Statistics and Mathematics, Zhejiang Gongshang University, Hangzhou, China; ^5^ Department of Hepatobiliary and Pancreatic Surgery, Affiliated Hangzhou First People’s Hospital, Zhejiang University School of Medicine, Hangzhou, China

**Keywords:** hepatocellular carcinoma, m6A-related lncRNA, tumor microenvironment, classification, prognosis, biomarkers, machine learning

## Abstract

HCC is one of the most common types of malignancies worldwide and the fourth-leading cause of cancer deaths. Thus, there is an urgent need to search for novel targeted therapies in HCC. 186 m6a-related lncRNAs were screened for subsequent analysis. Two distinct m6A modification clusters were identified to be associated with the overall prognosis in TCGA-LIHC based on the m6A-related lncRNAs profiling, followed by univariate Cox regression analysis. In addition, four m6A-related lncRNAs prognostic signatures were developed and validated that could predict the OS of HCC patients, followed by univariate Cox regression, LASSO regression, and multivariate Cox regression analysis. Moreover, four m6A-related lncRNAs were identified to be related to HCC prognosis. ESTIMATE was used to evaluate the stromal score, immune score, ESTIMATE score, and tumor purity of each HCC sample. ssGSEA was performed to identify the enrichment levels of 29 immune signatures in each sample. Finally, quantitative real-time polymerase chain reaction shown that KDM4A-AS1, BACE1-AS, and NRAV expressions were upregulated in HCC patients. We proved that our m6A-related lncRNAs signature had powerful and robust ability for predicting OS of different HCC subgroups.

## Introduction

Among primary liver cancer, HCC represents the major histological subtype. According to the global cancer statistics, HCC is one of the most common types of malignancies worldwide and the fourth leading cause of cancer deaths, causing approximately 66,2000 deaths each year worldwide (Venook et al., 2010; Ferlay et al., 2015). The incidence rates of HCC vary globally, but more than 80% of cases occur in low- and middle-resource countries, particularly in East Asia and Africa (Torre et al., 2015; Fitzmaurice et al., 2017; Yang et al., 2019). As one of the countries with the highest incidences of HCC in the world, China accounts for about 50% of newly diagnosed cases and deaths (Yang and Heimbach, 2020). Over the last few decades, intensive investigations and efforts have focused on the role of protein-coding genes in the pathogenesis of HCC to seek appropriate diagnostic markers and therapeutic targets for HCC (Hoshida et al., 2012; Xie et al., 2013). However, on a global scale, effective molecular markers for diagnosis, evaluation, and treatment of advanced HCC are still insufficient. Therefore, there is an urgent need to search for novel targeted therapies in HCC.

m6A is methylation that occurs in the N6-position of adenosine, which is the most prevalent epigenetic internal modification on eukaryotic messenger RNAs (mRNAs) and non-coding RNAs (ncRNAs) (He et al., 2019). m6A is installed by writers (methyltransferases), removed by erasers (demethylases), and recognized by readers (reader proteins) (Batista, 2017; Dai et al., 2018). In physiological processes, m6A regulates RNA metabolism, including translation, splicing, export, and degradation (Liu et al., 2017; Liu and Gregory, 2019). Accumulating evidence showed that m6A was involved in the pathogenesis and progression of cancer by regulating gene expression and affecting cell self-renewal, differentiation, invasion, and apoptosis (Deng et al., 2018; Panneerdoss et al., 2018; Karthiya and Khandelia, 2020; Lan et al., 2021). For instance, Niu et al. reported that FTO was overexpressed and promoted cell proliferation, colony formation, and metastasis in breast cancer by reducing BNIP3 methylation and promoting BNIP3 degradation (Niu et al., 2019). He et al. indicated that ALKBH5 was downregulated in pancreatic cancer with elevated m6A level and decreased expression of KCNK15-AS1, leading to enhanced migration and invasion of pancreatic tumor cells (He et al., 2018a). Meanwhile, increased research has suggested that m6A plays an important role in the occurrence and development of hepatocellular carcinoma, including METTL14 (Ma et al., 2017) and YTHDF2 (Zhong et al., 2019).

LncRNA is a non-coding RNA with a length of more than 200 nucleotides. Recent studies had shown that lncRNA played an important role in the pathophysiology of cancer, such as epigenetic regulation, cell cycle regulation, and cell regulation (Berretta and Morillon, 2009). It has been identified that the dysregulation of lncRNA is closely associated with tumorigenesis, metastasis, prognosis, and diagnosis in HCC (Abbastabar et al., 2018). For instance, overexpression of MALAT-1 could promote the proliferation, migration, and invasion of liver cancer cells by inhibiting the expression of mir-146-5p, thus leading to the recurrence and metastasis of hepatocellular carcinoma (Lai et al., 2012; Li et al., 2017).In addition, Wang et al. demonstrated that UCA1 might act as an endogenous sponge by repressing the expression of FGFR1 and activating an FGFR1/ERK signaling pathway in HCC (Wang et al., 2015). However, the role of m6A modification patterns in dysregulation of lncRNAs in HCC is still unclear.

In this study, we aimed to explore the m6A-related lncRNAs profiling and tumor microenvironment in HCC. Our study revealed two distinct subtypes based on m6A-related lncRNAs profiling and surprisingly found that the tumor microenvironment under these two clusters was highly consistent with overall survival, immunotherapy, and prognosis, respectively, suggesting that m6A-related lncRNAs played a pivotal and robust role in tumor microenvironment characterizations of individual HCC patients. For that, we established a novel m6A-related lncRNAs signature to predict prognosis and guide individual treatment strategies in HCC patients.

## Materials and Methods

### Data Source and m6A-Related lncRNAs

The mRNA expression files and clinical information of hepatocellular carcinoma were downloaded from TCGA database, including age, gender, tumor grade, pathologic stage, and AJCC-TNM. The annotation file of GRCH38 for long non-coding RNA was downloaded from GENCODE to identify lncRNA and mRNA. Based on the Ensemble IDs of the gene symbols, 14,086 lncRNAs were acquired in TCGA-LIHC. Furthermore, based on previous literature, the expression matrixes of 23 m 6A-related genes were obtained from TCGA-LIHC (Supplementary Table S1). Ultimately, we performed Pearson correlation analysis to obtain 186 m 6A-related lncRNAs for subsequent analysis with |Cor| > 0.5 and *p* < 0.001.

### Clustering, ESTIMATE, and ssGSEA

For TCGA-LIHC, we first performed univariate Cox regression analysis on the 186 m 6A-related lncRNAs to obtain 78 prognostic m6A-related lncRNAs. In addition, based on the 78 m 6A-related lncRNAs, we performed hierarchical clustering using the “ConsensusClusterPlus” package (R implementation, K = 2). ESTIMATE was used to evaluate the stromal score, immune score, ESTIMATE score, and tumor purity of each HCC sample (Yoshihara et al., 2013). ssGSEA was performed to identify the enrichment levels of 29 immune signatures in each sample (Barbie et al., 2009; Hänzelmann et al., 2013). 10-fold cross validation was implemented to evaluate the classification performance with the accuracy and the weighted F-score in TCGA-LIHC.

### M6A-Related lncRNAs Risk Signature in HCC

To identify the potential optimal m6A-related lncRNAs prognostic signature, we randomly divided TCGA-LIHC into two parts (training dataset and testing dataset). Followed by univariate Cox regression, LASSO regression, and multivariate Cox regression, we constructed a m6A-related lncRNA prognostic risk signature for HCC patients in the training dataset, which involved four m6A-related lncRNAs. The calculation formula of the risk score was: Risk score = Coef_AC099850.4_*Expression_AC099850.4_ + Coef_KDM4A-AS1_*Expression_KDM4A-AS1_+Coef_BACE1-AS_*Expression_BACE1-AS_ + Coef_NRAV_*Expression_NRAV_, where Coef represented coefficients with the lowest AIC values. A Coef greater than zero meant increased risk and *vice versa* to protective factors. In addition, we performed ROC curve and AUC values for the risk signature and other clinicopathological features to evaluate the prognostic ability in the training dataset. Similarly, we validated our risk signature in the testing dataset, followed by K-M survival analysis and ROC curve.

### Prediction Analysis of m6A-Related lncRNAs Risk Signature

To identify three of the four m6A-related lncRNAs expressions and their associations with HCC prognosis, the HCC tissues’ expressions and OS and DFS plots were downloaded from the GEPIA database. In addition, we put the training dataset and testing dataset together for subsequent analysis. All HCC patients were divided into high- or low-risk groups based on the median of risk scores as a threshold. We evaluated the risk scores in different clinicopathological features, followed by K-M survival analysis.

Additionally, the “limma” package was implemented to uncover the DEGs between the two subgroups. The “Metascape” website was employed for pathway and functional enrichment analysis involving GO Biological Process, KEGG pathway and Reactome Gene sets, and Canonical Pathways (Zhou et al., 2019). Finally, to identify the tumor hallmarkers, Gene Set Enrichment Analysis (GSEA) was performed.

### Four m6A-Related lncRNAs Function Prediction

To further identify how m6A-related lncRNAs regulated and influenced the development and progress of HCC, we performed co-expression analysis to predict downstream mRNAs with |Cor|> 0.5 and *p* < 0.001. 1985 related downstream mRNAs were identified and intersected with the DEGs to earn 97 m 6A-related lncRNAs targets. Cytoscape software 3.7.2 was implemented to visualize the co-expression networks. In addition, the “Metascape” online tool was used to perform functional and pathway enrichment analysis for the 97 m 6A-related lncRNAs targets.

### Cell Culture and Samples

To further evaluate the expressions of three of the four m6A-related lncRNAs in cells and tissues, L02, Huh7, HepG2, and Hep3B cells were cultured in DMEM containing 10% FBS. A total of 10 HCC samples and adjacent normal tissues were collected from HCC patients who underwent surgical resection from January 2018 to December 2020 in the Affiliated Hangzhou First People’s Hospital. All patients involved provided written informed consent. This research was approved by the Ethics Committee of Affiliated Hangzhou First People’s Hospital.

For assessment of three of the four m6A-related lncRNAs in cells and tissue, we extracted the total RNA using an RNA-Quick purification kit. A PrimeScriptTM RT reagent kit with gDNA Eraser (Takara, RR047A) was employed to reverse the total RNA into cDNA. Quantitative real-time polymerase chain reaction was conducted using TB Green Premix EX Taq^TM^ II (Takara, RB820A). M6A-related lncRNAs expressions were analyzed using the 2^−ΔΔC^ method (Table 1).

**TABLE 1:** Primers’ sequences.

Genes’ names	Primers	Sequences (5’ → 3′)
KDM4A-AS1	Forward	CAG​GGA​AGA​GTG​AAC​CAG​GAC​AAA​G
	Reverse	CTA​GAA​GCA​GGA​GAG​GGC​AGA​GAT​AG
BACE1-AS	Forward	TGG​CTG​TTG​CTG​AAG​AAT​GTG​ACT​C
	Reverse	CAA​CCT​TCG​TTT​GCC​CAA​GAA​AGT​G
NRAV	Forward	CTA​ATA​GTC​AAG​CAC​CGC​CTG​AGT​AG
	Reverse	CTG​TTA​GTC​CTC​TCT​GGT​TGT​GTC​TTC
*β*-actin	Forward	ATC​GTG​CGT​GAC​ATT​AAG​GAG​AAG
	Reverse	AGG​AAG​GAA​GGC​TGG​AAG​AGT​G

### Statistical Analysis

All calculations and analysis were implemented using R software 4.0.4 and Perl language. Kaplan–Meier survival analysis and log-rank test were employed to compare overall survival of two subgroups. The “limma” package was used to generate differential expressed genes. The “ConsensusClusterPlus” package was performed for hierarchical clustering analysis. Followed by univariate Cox regression, LASSO regression, and multivariate Cox regression, a m6A-related risk signature was constructed and validated. Independent prognostic factors were identified with univariate Cox regression and multivariate Cox regression analysis. Student’s *t*-test was performed for statistical comparisons. Values of *p* < 0.05 were considered statistically significant.

## Results

### Identification of m6A-Related lncRNAs in TGCA-LIHC

**FIGURE 1:**
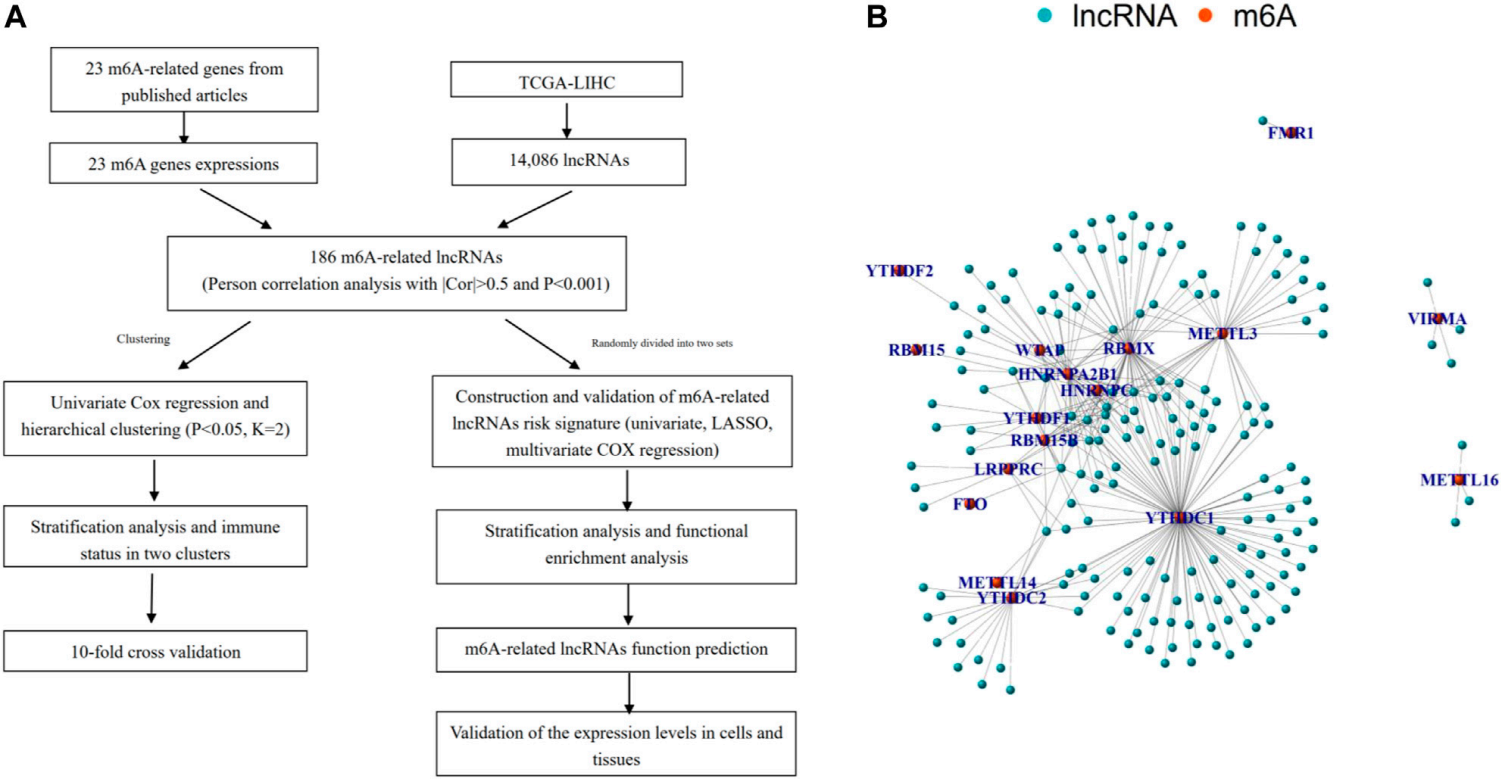
Overview of study. **(A)** Study flow chart. **(B)** Network of the correlations between m6A-related genes and 186 m6A-related lncRNAs.

First, we downloaded the expression matrixes of 374 HCC samples and 50 matched normal controls from TCGA database. Using the annotation file downloaded from the GENCODE website, we confirmed 14,806 lncRNAs and 19,604 mRNAs for subsequent analysis. Then the expression matrixes of 23 m 6A-related genes were extracted and analyzed from the TCGA-LIHC cohort. An m6A-related lncRNA was defined as a lncRNA associated with one or more of 23 m 6A-related genes using Pearson correlation analysis with |Cor| > 0.5 and *p* < 0.001. The workflow of the entire research is shown in Figure 1A, and the associations of 23 m 6A-related genes and 186 lncRNAs are shown in Figure 1B.

### Hierarchical Clustering Identifies Two HCC Subtypes Based on m6A-Related lncRNAs Profiling

**FIGURE 2:**
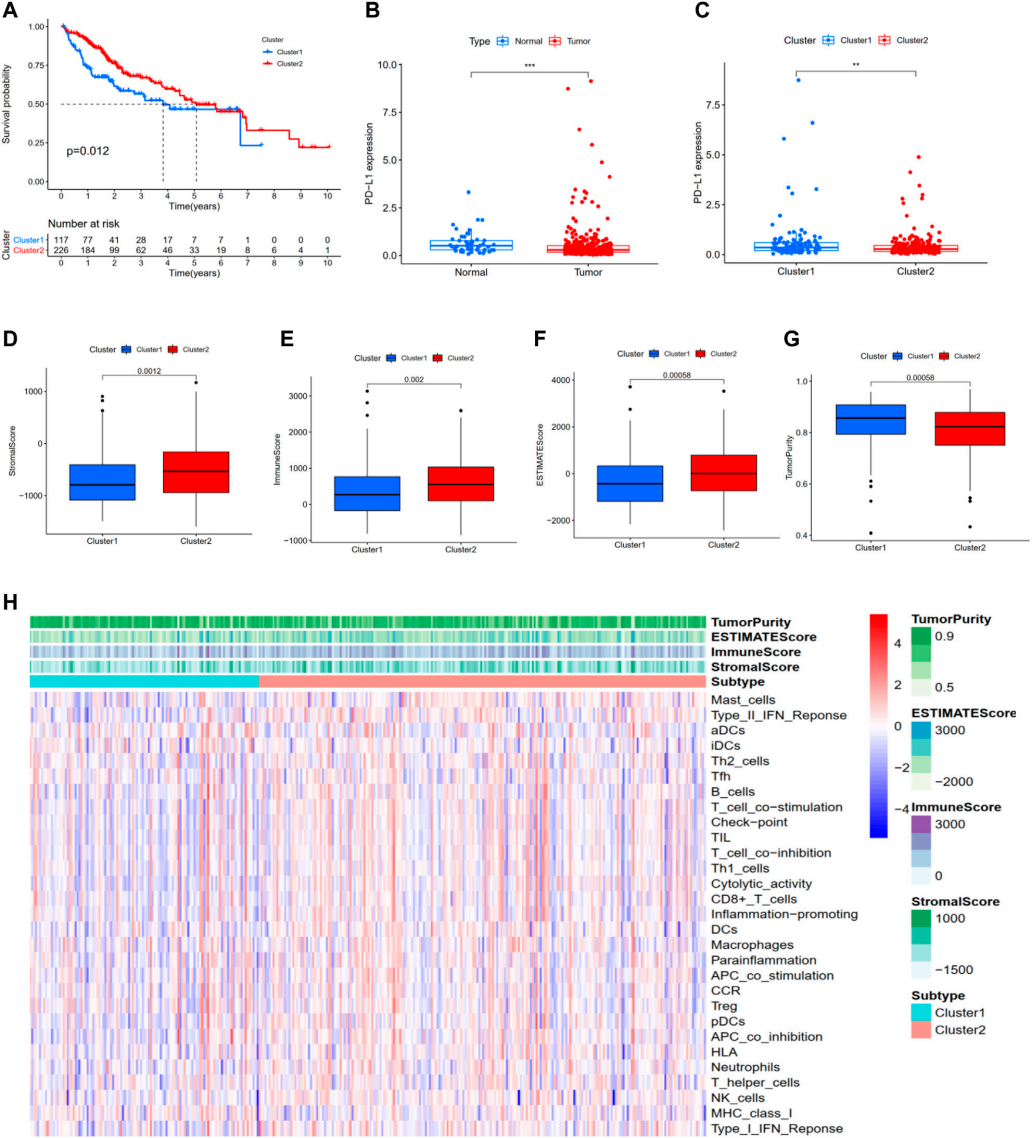
Hierarchical clustering identifies two HCC subtypes based on m6A-related lncRNAs profiling. **(A)** Kaplan–Meier survival analysis illustrated that Cluster 2 (Immune Response High) was significantly associated with better prognosis. **(B,C)** PD-L1 was more highly expressed in tumor and Cluster one than in normal and Cluster 2. **(D)** Stromal score. **(E)** Immune score. **(F)** ESTIMATE score. **(G)** Tumor purity. **(H)** Heatmap of the enrichment levels of 29 immune signatures in each sample. *p < 0.05; **p < 0.01; ***p < 0.001.

For the previously obtained 186 m 6A-related lncRNAs, we performed univariate Cox regression analysis and identified 78 m 6A-related prognostic lncRNAs. On the basis of the 78 m 6A-related lncRNAs, we hierarchically clustered HCC using the “ConsensusClusterPlus” package (R implementation, K = 2). Interesting, HCC patients were clearly separated into two clusters. We defined the two clusters as Cluster 1 (Immune Response Low) and Cluster 2 (Immune Response High), respectively. K-M survival analysis showed that Cluster two was significantly associated with better prognosis (*p* = 0.012, Figure 2A). In addition, PD-L1 was more highly expressed in tumor and Cluster one than in normal and Cluster 1: PD-L1 might act as a mediator in tumor progression (Figures 2B,C). We performed ESTIMATE to evaluate the stromal score, immune score, ESTIMATE score, and tumor purity of each HCC sample. When comparing the stromal score, immune score, and ESTIMATE score in two clusters, we found scores increasing from Cluster one to Cluster 2 (Figures 2D–F). Tumor purity had the opposite trend (Figure 2G). Above all, these results demonstrated that Cluster two had a higher number of immune cells and stromal cells, while Cluster one contained more tumor cells.

Moreover, as shown in Figure 2H, ssGSEA was performed to identify the enrichment levels of 29 immune signatures in each sample. 10-fold cross validation was implemented to evaluate the classification performance with an accuracy value of 91% and F-score of 89%. Collectively, these results proved that hierarchical clustering identifies two subtypes significantly associated with the overall prognosis in TCGA-LIHC based on the m6A-related lncRNAs profiling.

### Construction and Validation of Four m6A-Related lncRNAs Prognostic Risk Signature in TCGA-LIHC

**FIGURE 3:**
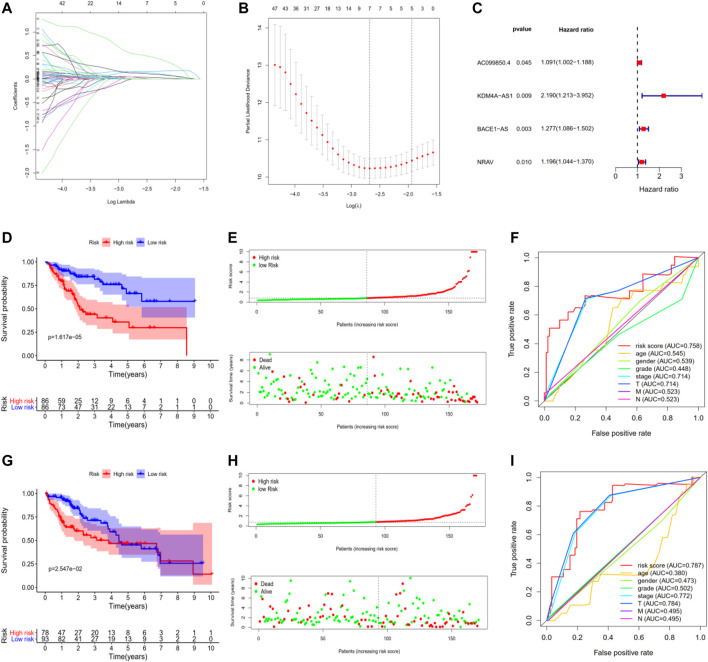
Construction and validation of four m6A-related lncRNAs prognostic risk signature in TCGA-LIHC. **(A,B)** LASSO regression was performed, calculating the optimal criteria. **(C)** Coefficients. **(D)** Kaplan–Meier survival analysis showed that the high-risk group had poor prognosis and shorter overall survival in the training set. **(E)** The scatter plot of risk scores in the training set. **(F)** The scatter plot of survival status in the training set. **(G–I)** The survival plot, scatter plot of risk scores, and survival status in the testing set.

To construct a potential optimal m6A-related lncRNA to predict the overall survival of HCC patients, we randomly divided TCGA-LIHC into two parts, namely, the training set and the testing set (Supplementary Tables S2,3). For the training set, we constructed a m6A-related lncRNAs prognostic signature based on the 186 m 6A-related lncRNAs, which involved Coefs of each and four m6A-related lncRNAs (Figures 3A,B). We calculated the risk scores of each HCC patient based on coefficients and gene expression (Figure 3C, Supplementary Figure S2; Table 2). We divided the HCC patients of the training set into high- and low-risk subgroups based on the median of risk scores as the threshold. K-M survival analysis showed that HCC patients with high-risk scores suffered shorter overall survival and worse prognosis (Figure 3D). The survival status distribution proved that HCC patients died more as the risk increased (Figure 3E). In addition, we evaluated the prognostic ability of the risk signature compared with age, gender, tumor grade, pathologic stage, and AJCC-TNM. The ROC curve analysis showed that our four m6A-related lncRNA had the optimal prognostic ability to forecast the OS of HCC patients (AUC = 0.758, Figure 3F).

**TABLE 2:** Multivariate Cox regression analysis results during signature construction.

LncRNAs	HR (95% CI)	*p*-value	Coef
AC099850.4	1.091 (1.002–1.188)	0.045	0.087
KMD4A-AS1	2.190 (1.213–3.952)	0.009	0.784
BACE1-AS	1.277 (1.086–1.502)	0.003	0.245
NRAV	1.196 (1.044–1.370)	0.010	0.179

HR, hazard ratio; CI, confidence interval; Coef = regression coefficient.

To validate our m6A-related lncRNAs prognostic signature, we calculated the risk scores of each HCC patient in the testing set using the same scheme. The results were similar with the training set: HCC patients with high risk had worse survival outcome with statistical significance (Figure 3G, Supplementary Figure S2). The distributions of risk scores and survival status were shown in Figure 3H. ROC curve analysis demonstrated that the risk score has a stable and robust predictive ability (AUC = 0.787, Figure 3I). All results indicated that our four m6A-related lncRNAs signatures could predict the OS of HCC patients.

### Three of Four m6A-Related lncRNAs Expressions and Their Relationships With Prognosis in HCC Patients

**FIGURE 4:**
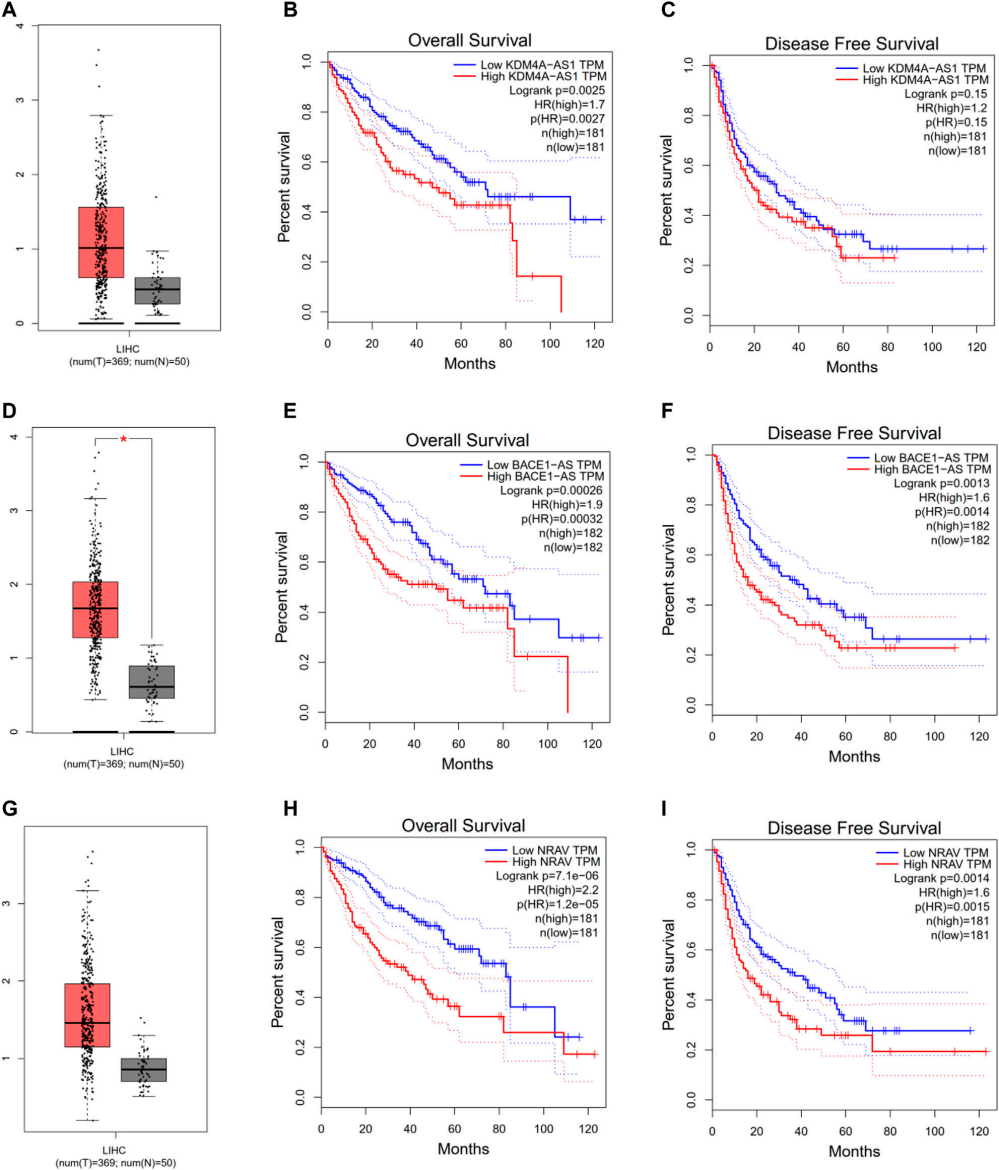
Evaluating three of the four m6A-related lncRNAs in the GEPIA database. **(A–C)** KDM4A-AS1. **(D–F)** BACE1-AS. **(G–I)** NRAV expression, overall survival plot, and disease-free survival plot from the GEPIA database, respectively.

To further explore three of the four m6A-related lncRNAs expressions and their associations with prognosis in HCC, the Gepia database was employed to obtain the gene expressions, overall survival, and disease-free survival of three m6a-related lncRNAs in HCC. As shown in Figure 4, compared with the control group, KDM4A-AS1, BACE1-AS, and NRAV were significantly elevated in HCC (Figures 4A–G). Additionally, HCC patients with increased expressions of KDM4A-AS1, BACE1-AS, and NRAV had shorter OS and DFS, as well as worse prognosis (Figures 4B–I). Thus, these results suggested that three of four m6A-related lncRNAs could be implemented as independent biomarkers for predicting prognosis in HCC.

### Association Between Risk Signature and Other Clinicopathological Features in TCGA-LIHC

**FIGURE 5:**
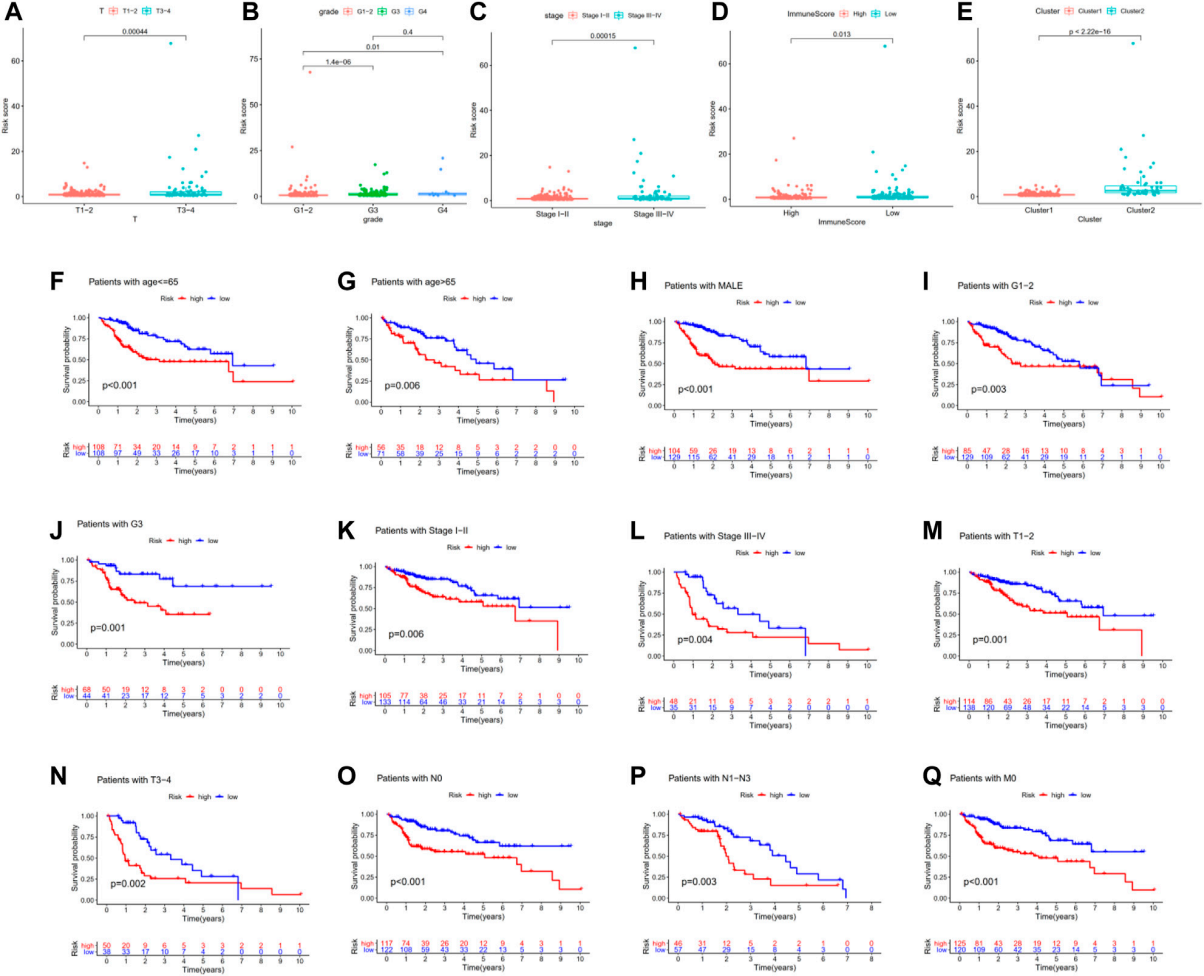
Association between risk signature and other clinicopathological features in TCGA-LIHC. **(A–E)** Risk scores were associated with AJCC-T, tumor grade, pathologic stage, immune score, and hierarchical clusters. **(F–Q)** Compared with low-risk patients, high-risk HCC patients in various subgroups had shorter OS and worse prognosis.

We attempted to explore the associations between risk signature and other clinicopathological features in TCGA-LIHC. The results indicated that risk scores were associated with AJCC-T, tumor grade, pathologic stage, immune score, and hierarchical clusters (Figures 5A–E). To better evaluate the independent prognostic ability of m6A-related lncRNAs, we conducted a stratified analysis to confirm whether m6A-related lncRNAs could predict OS of different HCC subgroups. Compared with low-risk patients, high-risk HCC patients in various subgroups (age<65, age≥65, male, G1-2, G3, Stage I-II, Stage III-IV, T1-2, T3-4, N0, N1-3 and M0 subgroups) had shorter OS and worse prognosis (Figure 5F–Q).

Moreover, univariate Cox regression analysis indicated that pathologic stage, AJCC-T, and risk score were associated with the prognosis of HCC patients. Multivariate Cox regression analysis proved that risk score is an independent prognostic indicator for HCC patients (Table 3). These data proved that our m6A-related lncRNAs signature had powerful and robust ability for predicting OS of different HCC subgroups.

**TABLE 3:** Univariate and multivariate Cox regression analysis of OS in TCGA-LIHC.

Clinicopathologic parameters	Univariate analysis		Multivariate analysis	
	HR (95%CI)	*P*	HR (95%CI)	*P*
Age	0.998 (0.980–1.016)	0.827		
Gender	0.768 (0.471–1.251)	0.289		
Tumor grade	1.019 (0.737–1.407)	0.913		
Pathologic stage	2.095 (1.612–2.723)	<0.001*	1.055 (0.382–2915)	0.918
AJCC-T	2.006 (1.575–2.554)	<0.001*	1.796 (0.715–4.513)	0.213
AJCC-N	2.305 (0.562–9.458)	0.246		
AJCC-M	4.386 (1.371–14.038)	0.013*	1.505 (0.393–5.759)	0.551
Risk score	1.129 (1.079–1.181)	<0.001*	1.095 (1.040–1.152)	<0.001*

TNM, tumor-node-metastasis; HR, hazard ratio; *p < 0.05 was considered statistically significant.

### Functional Enrichment and GSEA

**FIGURE 6:**
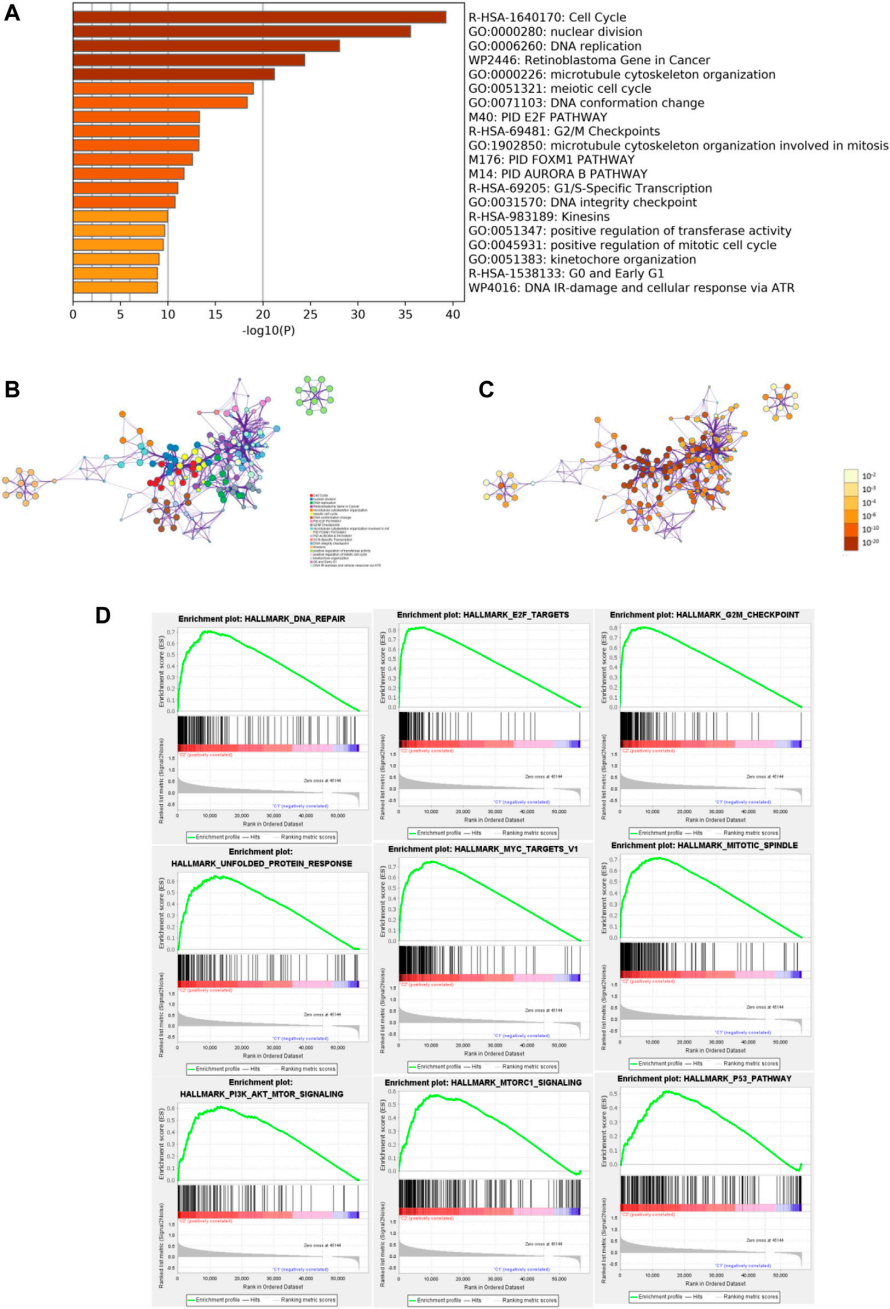
Functional enrichment and Gene Set Enrichment Analysis (GSEA). **(A)** Heatmap of enriched terms between high- and low-risk groups, colored according to *p*-value. Network of enriched terms colored according to **(B)** cluster ID and **(C)** *p*-value. **(D)** Gene Set Enrichment Analysis (GSEA) showed that tumor hallmarkers were enriched in the high-risk group.

To identify the potential functional and pathway enrichment involved in gene heterogeneity between high- and low-risk subgroups, R package “limma” was employed to obtain 2054 DEGs, followed by the standards of *p* < 0.05 and |log2(Foldchange)| > 1. We put 2054 DEGs into the “Metascape” online tool for analysis, which mainly enriched in these terms: Cell Cycle, nuclear division, DNA replication, Retinoblastoma Gene in Cancer, microtubule cytoskeleton organization, and so on (Figures 6A–C). Gene Set Enrichment Analysis (GSEA) was performed to identify the tumor hallmarkers enriched in the high-risk group, such as PI3K-AKT-mTOR signaling, mTORC1 signaling, the P53 pathway, and so on (Figure 6D). These results could provide us with novel insights into molecular biological function related to m6A-related lncRNAs.

### Predicting m6A-Related lncRNAs Function Using Co-Expression Analysis

**FIGURE 7:**
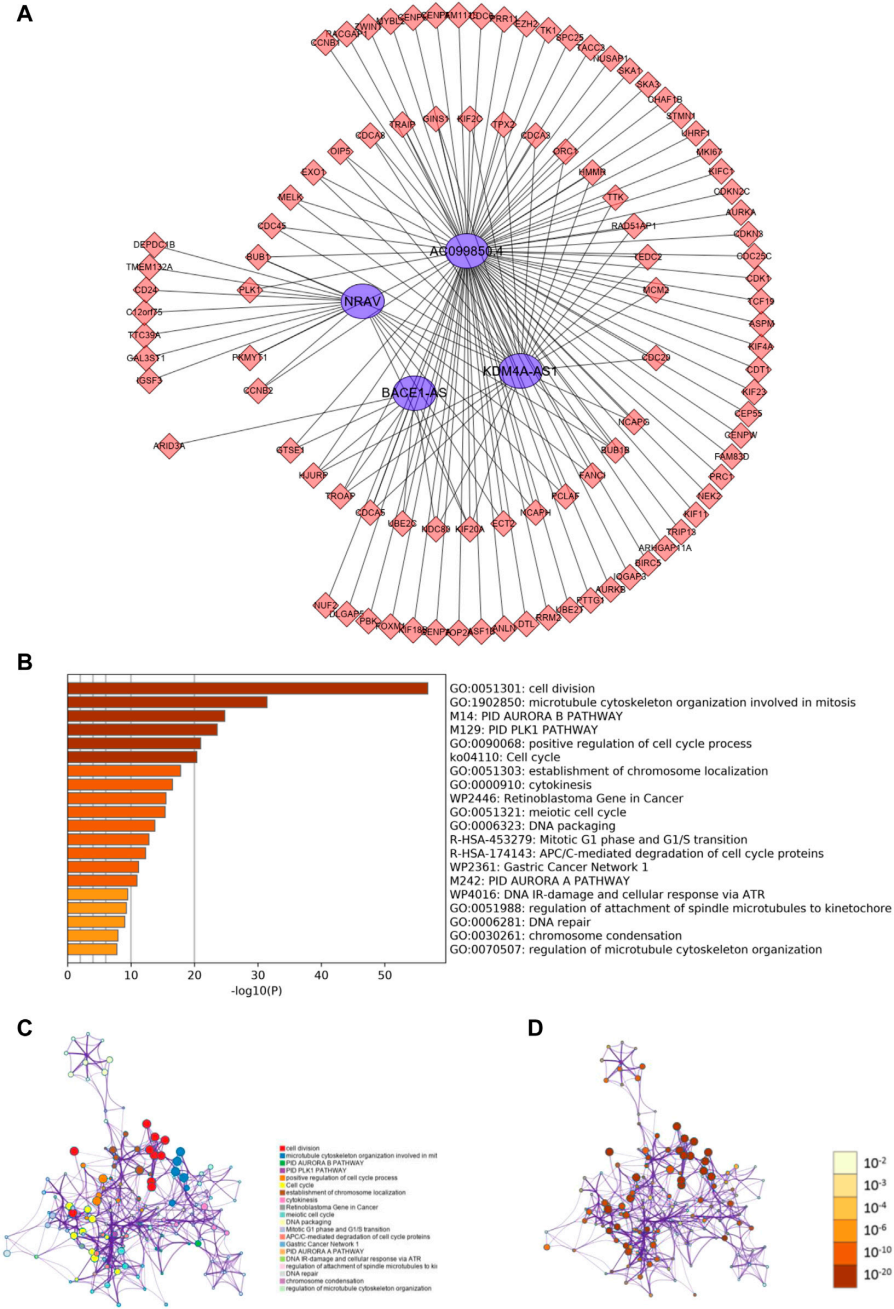
Predicting m6A-related lncRNAs function using co-expression analysis. **(A)** Network of four m6A-related lncRNAs and their target mRNA using co-expression analysis. **(B)** Heatmap of enriched terms across the 97 target mRNAs. Network of enriched terms across the 97 target mRNAs colored according to **(B)** cluster ID and **(C)** *p*-value.

To investigate how m6A-related lncRNAs regulate and influence the development and progress of HCC, we performed co-expression analysis to predict downstream mRNAs with |Cor|> 0.5 and *p* < 0.001. 1985 related downstream mRNAs were identified and intersected with the DEGs to earn 97 m 6A-related lncRNAs targets (Figure 7A). Metascape was performed for functional and pathway enrichment analysis of 97 mRNAs. The enriched terms were cell division, microtubule cytoskeleton organization involved in mitosis, PID AURORA B PATHWAY, PID PLK1 PATHWAY, and so on. These data mainly provide us with clues to confirm the functions of the four m6A-related lncRNAs.

### Validation of Three m6A-Related lncRNAs Expression in Cells and Tissues

**FIGURE 8:**
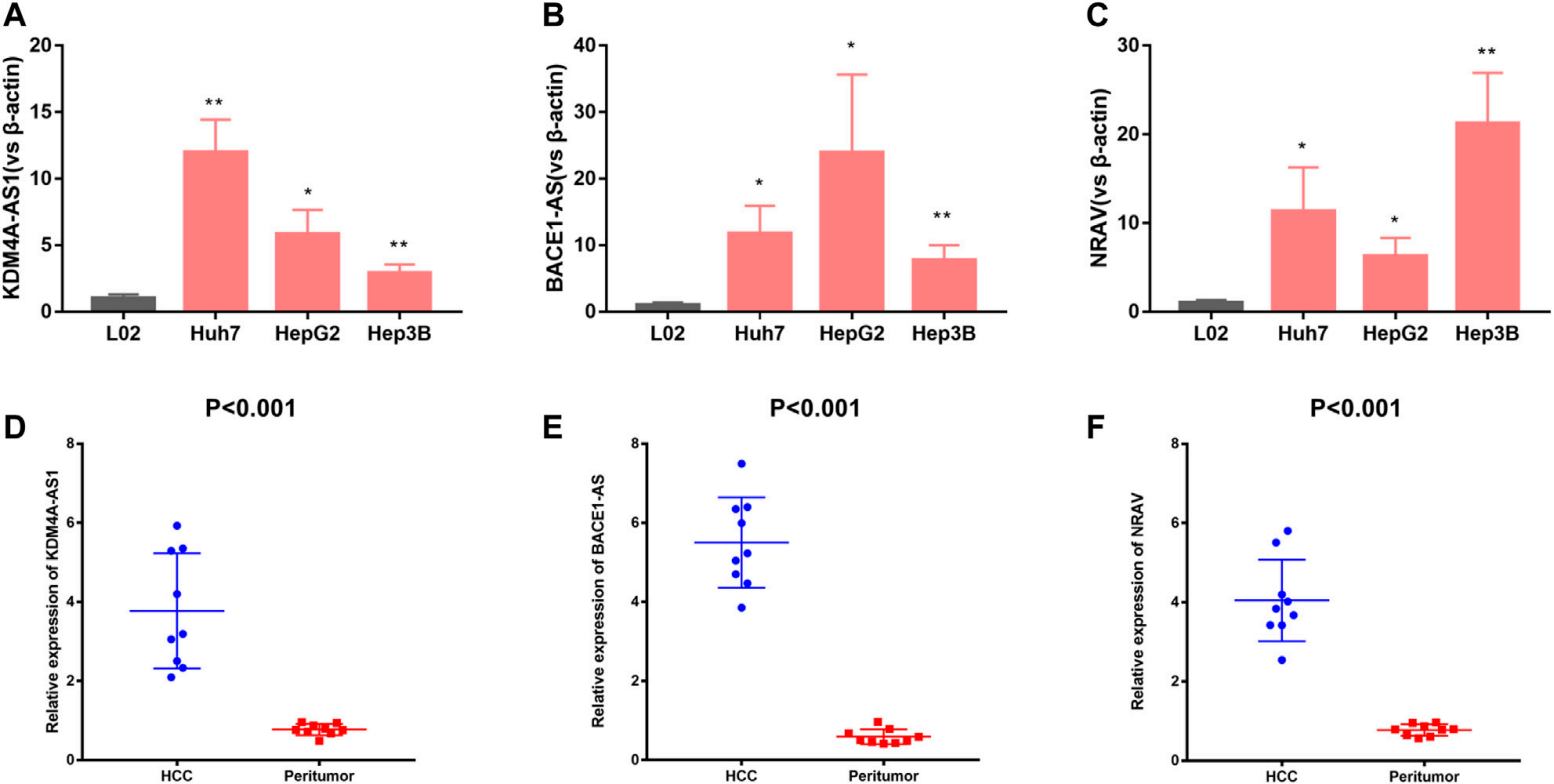
Validation of three m6A-related lncRNAs expression in cells and tissues. **(A–C)** KDM4A-AS1, BACE1-AS, and NRAV expression in normal liver cells and HCC cells. **(D–F)** KDM4A-AS1, BACE1-AS, and NRAV expression in HCC tissues and adjacent normal tissues.

For validation of three of the four m6A-related lncRNAs expressions in cells and tissue, we detected three of the four m6A-related lncRNAs expressions in L02, Huh7, HepG2, and Hep3B cells. The results showed that KDM4A-AS1, BACE1-AS, and NRAV expressions were significantly upregulated in HCC cells in contrast to normal liver cells (Figures 8A–C). In addition, we detected three m6A-related lncRNAs expressions in 10 pairs of HCC tissues and adjacent normal tissues we collected by quantitative real-time polymerase chain reaction (Q-PCR). Our results indicated that KDM4A-AS1, BACE1-AS, and NRAV expressions were upregulated in HCC patients (Figures 8D–F).

## Discussion

A total of 374 HCC samples from TCGA database were included in our study to explore the m6A-related lncRNAs profiling and tumor microenvironment. 186 m 6A-related lnRNAs were obtained from TCGA-LIHC. Two HCC subtypes were confirmed by hierarchical clustering on the basis of 186 m 6A-related lncRNAs, followed by univariate Cox regression analysis, which were associated with the OS and tumor microenvironment. For that, the TCGA-LIHC database was randomly separated into the training dataset and the testing dataset. Four m6A-related lncRNAs prognostic risk signatures were constructed and validated in TCGA-LIHC. In addition, we performed co-expression analysis to identify the potential function of the four m6A-related lncRNAs. Finally, Q-PCR was implemented to detect three of the four m6A-related lncRNAs expressions in cells and tissue.

Abundant evidence had suggested that m6A modification was involved in cancer pathogenesis and progression with epigenetic regulation of oncogenes or tumor suppressor genes (He et al., 2018b). However, how it works in a lncRNA-dependent manner during hepatocellular carcinoma is still unknown. m6A modification can regulate malignance and invasion of several tumors by modifying specific lncRNAs. Yuan et al. reported that overexpressing METTL3 significantly promoted osteogenic differentiation of primary ligament fibroblasts by increasing m6A methylation of long non-coding RNA (lncRNA) X-inactive specific transcript (XIST) (Yuan et al., 2021). M6A reader YTHDF3 negatively regulates the long non-coding RNA GAS5, inhibiting the progression of colorectal cancer by interacting with and triggering YAP phosphorylation and degradation (Ni et al., 2019). Furthermore, Yang et al. indicated that low expression of TRAF3IP2-AS1 promotes the progression of NONO-TFE3 translocation renal cell carcinoma by stimulating the N6-methyladenosine of PARP1 mRNA and downregulating PTEN (Yang et al., 2021). Meng et al. demonstrated that m6A-mediated upregulation of LINC00857 promotes the occurrence of pancreatic cancer by functioning as a competing endogenous RNA (ceRNA) for sponging miR-150-5p, leading to overexpressing E2F3 and ultimately promoting tumorigenesis in PC (Meng et al., 2021) Studies had shown that the m6A modification of lncRNA may affect cancer occurrence and development, and lncRNA could act as a competitive endogenous RNA to target m6A modulators, thereby affecting tumor invasion and progression. Taken together, we firmly believe that m6A modification could target lncRNA to regulate the occurrence and development of tumors, and we should pay more attention to the function and interaction between m6A modification and lncRNA to identify potential prognostic markers or therapeutic targets for cancers.

In this study, four m6A-related lncRNAs were identified to be related to HCC prognosis, and a few studies had revealed biological functions of these lncRNAs. Elevation of lncRNA BACE1-AS was a potential mechanism to inhibit the proliferation and invasion of human ovarian cancer stem cells and may be a novel target of anisomycin in the treatment of ovarian cancer (Chen et al., 2016). LncRNA NRAV negatively regulated the initial transcription of multiple key interferon-stimulated genes and was important in regulating the antiviral interferon response (Ouyang et al., 2014). In the meantime, lncRNA NRAV has also been proven to be an independent prognostic factor for patients with low-grade glioma (Maimaiti et al., 2021). A recent meta-analysis reported that KDM4A-AS1 was screened as an important prognostic factor to predict overall survival in HCC patients. However, there were few reports on the modification and interaction between lncRNAs and m6A-related genes in HCC. Thus, we hope that our results would help identify m6a-related prognostic lncRNAs, thereby providing novel insights into their potential role and serving as novel therapeutic targets in HCC.

However, there were several limitations and challenges in our study. The modification and interaction between lncRNA and m6A-related genes should be confirmed by *in vivo* and *in vitro* experiments. Indeed, it is essential to further investigate the molecular mechanisms and signaling pathways of m6A-related lncRNAs involved in HCC.

In conclusion, our study revealed two distinct subtypes based on m6A-related lncRNAs profiling and identified a novel m6A-related lncRNAs signature to predict prognosis and guide individual treatment strategies in HCC patients.

## Data Availability

The datasets presented in this study can be found in online repositories. The names of the repository/repositories and accession number(s) can be found in the article/Supplementary Material.

## Abbreviations

AUC, area under the curve; HCC, hepatocellular carcinoma; HR, hazard ratio; m6A-related lnRNAs, N6-Methylandenosine-Related lncRNAs; OS, overall survival; ROC curve, receiver operating characteristic curve; ssGSEA, single sample gene set enrichment analysis; TCGA, The Cancer Genome Atlas; TNM: tumor-node-metastasis.
