# Supplementary material for: Classification of Hepatocellular Carcinoma Based on N6-Methylandenosine–Related lncRNAs Profiling
Source: Front Mol Biosci. 2022 Feb 4;9:807418. doi: 10.3389/fmolb.2022.807418 (PMC8854774; doi:10.3389/fmolb.2022.807418)
Supplement: Supplementary file 1 [file Table5.DOCX]

**Table S2. Basic clinical information of 172 HCC patients in training set.**

| **Variables** | **Training set**  **(n=172)** |
| --- | --- |
| Age | 60.26±13.45 |
| Gender  Female  Male | 60(34.9%)  112(65.1%) |
| Tumor Grade  G1&G2  G3&G4  Unknow | 102(59.3%)  68(39.5%)  2(1.2%) |
| Pathologic Stage  I&II  III&IV  Unknow | 114(66.3%)  46(26.7%)  12(7.0%) |
| AJCC-T  T1  T2  T3  T4  Unknow | 78(45.3%)  43(25.0%)  41(23.8%)  8(4.7%)  2(1.2%) |
| AJCC-N  N0  N1-N3  Unknow | 116(67.4%)  2(1.2%)  54(31.4%) |
| AJCC-M  M0  M1  Unknow | 124(72.1%)  2(1.2%)  46(26.7%) |

Values are mean ± standard deviation or n (%).
