# Supplementary material for: Classification of Hepatocellular Carcinoma Based on N6-Methylandenosine–Related lncRNAs Profiling
Source: Front Mol Biosci. 2022 Feb 4;9:807418. doi: 10.3389/fmolb.2022.807418 (PMC8854774; doi:10.3389/fmolb.2022.807418)
Supplement: Supplementary file 2 [file Table4.DOCX]

**Table S1. 23 m6A-related genes from published literature.**

| **Genes** | **Types** |
| --- | --- |
| METTL3 | writers |
| METTL14 | writers |
| METTL16 | writers |
| WTAP | writers |
| VIRMA | writers |
| ZC3H13 | writers |
| RBM15 | writers |
| RBM15B | writers |
| YTHDC1 | readers |
| YTHDC2 | readers |
| YTHDF1 | readers |
| YTHDF2 | readers |
| YTHDF3 | readers |
| HNRNPC | readers |
| FMR1 | readers |
| LRPPRC | readers |
| HNRNPA2B1 | readers |
| IGF2BP1 | readers |
| IGF2BP2 | readers |
| IGF2BP3 | readers |
| RBMX | readers |
| FTO | erasers |
| ALKBH5 | erasers |
