# Supplementary material for: Classification of Hepatocellular Carcinoma Based on N6-Methylandenosine–Related lncRNAs Profiling
Source: Front Mol Biosci. 2022 Feb 4;9:807418. doi: 10.3389/fmolb.2022.807418 (PMC8854774; doi:10.3389/fmolb.2022.807418)
Supplement: Supplementary file 3 [file Table6.DOCX]

**Table S3. Basic clinical information of 171 HCC patients in testing set.**

| **Variables** | **Testing set**  **(n=171)** |
| --- | --- |
| Age | 58.53±13.01 |
| Gender  Female  Male | 50(29.2%)  121(70.8%) |
| Tumor Grade  G1&G2  G3&G4  Unknow | 112(65.5%)  56(32.7%)  3(1.8%) |
| Pathologic Stage  I&II  III&IV  Unknow | 124(72.6%)  37(21.6%)  10(5.8%) |
| AJCC-T  T1  T2  T3  T4  Unknow | 90(52.6%)  41(24.0%)  34(19.9%)  5(2.9%)  1(0.6%) |
| AJCC-N  N0  N1-N3  Unknow | 123(71.9%)  1(0.6%)  47(27.5%) |
| AJCC-M  M0  M1  Unknow | 121(70.8%)  1(0.6%)  49(28.7%) |

Values are mean ± standard deviation or n (%).
